# *Lissonema sicki*, an emerging air sac nematode of European owls: introduction, host switching and rapid establishment on a Mediterranean island

**DOI:** 10.1017/S0031182024000805

**Published:** 2024-07

**Authors:** Sebastià Jaume-Ramis, Sofía Delgado-Serra, Jordi Miquel, Nieves Negre, Ugo Mameli, Carles Feliu, Claudia Paredes-Esquivel

**Affiliations:** 1 Mediterranean Parasitology and Ecoepidemiology Research Group, University of the Balearic Islands, Mallorca, Spain; 2 Secció de Parasitologia, Departament de Biologia, Sanitat i Medi Ambient, Facultat de Farmàcia i Ciències de l'Alimentació, Universitat de Barcelona, Barcelona, Spain; 3 Institut de Recerca de la Biodiversitat (IRBio), Universitat de Barcelona, Barcelona, Spain; 4 Consortium for the Recovery of Wildlife of the Balearic Islands (COFIB), Mallorca, Spain; 5 CIBER Enfermedades Infecciosas CIBERINFEC-MICINN-ISCIII, Madrid, Spain

**Keywords:** air sacs, Aproctidae, helminths, invasive parasites, *Lissonema sicki*, Mallorca, Strigiformes

## Abstract

In recent years, air sac parasitic helminths have been reported to cause severe disease in birds. In addition, various species appear to be expanding and infecting new avian hosts in various regions worldwide. In this context, an air sac nematode was initially detected in 2014 infecting the Eurasian scops owl, hospitalized in the local wildlife hospital in Mallorca (Balearic Islands, Spain). Years later, the parasite was detected in 2 other owl species. Air sac nematodes had never been reported in the Mallorcan Strigiformes before. A comprehensive molecular and morphological characterization analysis, including scanning electron microscopy, was required for species confirmation. The species was identified as *Lissonema sicki*, a parasite infrequently reported in South American owls. Since its first introduction to Mallorca, it has dramatically increased in prevalence in hospitalized birds, being highly prevalent in the Eurasian scops owl (41%), in the long-eared owl (11%) and in the barn owl (4%). The introduction pathway of this parasite to Europe remains unknown. This discovery underscores the expanding range and impact of *L. sicki*, emphasizing the importance of ongoing surveillance and research to comprehend and manage the implications of its emergence in new territories.

## Graphical abstract

**Figure:**
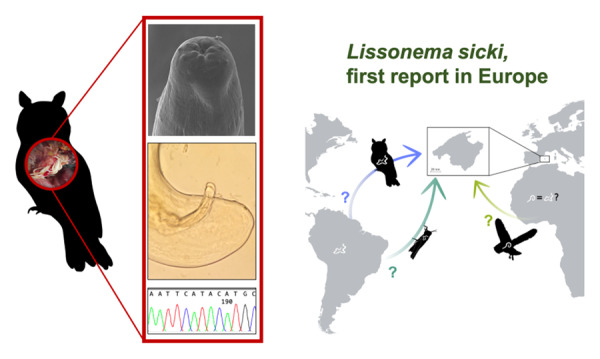

## Introduction

Parasitic infections have the potential to cause severe disease outbreaks in birds. Although mild infections tend to be asymptomatic (Höfle *et al*., 2003; Liptovszky *et al*., 2012; Galosi *et al*., 2019), high parasitic loads can negatively impact hosts' population dynamics by decreasing body mass, nesting success, fecundity and clutch size, increasing nestling mortality (Hamilton and Marlene, 1982; Møller *et al*., 1990, 2013; Loye and Zuk, 1991; Marzal *et al*., 2005; Brym *et al*., 2018). Helminth parasites are generally considered to be less pathogenic than protozoans; however, virulence varies widely depending on the parasite group and the specific interaction with their hosts (Gutiérrez *et al*., 2019).

In birds, global change is directly impacting the dynamics of parasitic infections (Hakalahti *et al*., 2006; Garamszegi, 2011). Climatic variations can induce alterations of migration routes, resulting in the introduction of new parasites into previously unoccupied regions (Harvell *et al*., 2002; Cattadori *et al*., 2005; Brooks and Hoberg, 2007). Furthermore, episodes of parasite host switching, which appear to be widespread, are believed to be influenced by global change (Brooks and Hoberg, 2007; Hoberg and Brooks, 2008). These changes in susceptible host species may eventually result in vulnerable or endemic birds contracting novel parasites, which can lead to population declines or even extinctions (Fuller *et al*., 2012). Therefore, having a comprehensive understanding of the parasites circulating among endemic and migrant birds is crucial to assess the potential for outbreaks. In Europe, long-term monitoring of bird populations has revealed a steady decline in species diversity (European Environment Agency, 2020), along with changes in bird dynamics (Zalakevicius, 2000; Crick, 2004), which may also have a negative impact on helminth diversity (Sitko and Heneberg, 2020*b*, 2021). Moreover, the observation of uncommon bird species has become increasingly frequent in the Balearic Islands (Garcia-Febrero *et al*., 2011; López-Jurado *et al*., 2020).

Among bird helminths, those that infect air sacs have been historically overlooked, because they have not been associated with major health issues (Cooper, 1969). Furthermore, their correct identification is challenging given the scarcity of updated and available keys as well as the limited number of species studied using a molecular approach. Although these parasites tend to cause asymptomatic infections, an increasing number of reports have shown that they can also cause severe clinical manifestations and even lead to death (Dronen *et al*., 2009; Galosi *et al*., 2019; Díaz *et al*., 2022). Some of these helminths appear to be expanding their distribution or infecting new avian hosts. One clear example is the nematode *Serratospiculum tendo* (Nitzsch, 1819) of the family Diplotriaenidae which has recently been reported in Peru and Argentina and is expanding across South America among Austral peregrine falcons (Order Falconiformes, *Falco peregrinus* Tunstall, 1771) (Gomez-Puerta *et al*., 2014; Ibarra *et al*., 2019) and the diplotriaenid nematode *Serratospiculoides amaculata* (Wehr, 1938) which has been also increasing its geographic range and switched host to passerines (Abdu *et al*., 2023). In the USA, there has been a growing number of reports of air sac trematodes from the family Cyclocoelidae in zoos (Dronen *et al*., 2009). In Europe, a comprehensive spatiotemporal study in the common blackbird (Passeriformes, *Turdus merula* Linnaeus, 1758) showed that the cyclocoelid air sac trematode *Morishitium polonicum* (Machalska, 1980) was absent in this bird species prior 1990 and its prevalence has increased, reaching 19% in recent years (Sitko and Heneberg, 2020*a*). This same species was reported from the air sacs of a song thrush (Passeriformes, *Turdus philomelos* Brehm, 1831) in the Balearic Islands (Jaume-Ramis and Pinya, 2018).

The primary objective of this study was to conduct a comprehensive morphological, ultrastructural and molecular characterization of nematodes infecting the air sacs of strigiform birds that entered the wildlife hospital and to analyse prevailing trends in their prevalence. Furthermore, we engage in a critical evaluation of their role as emerging pathogens within the affected bird population.

## Materials and methods

The Balearic archipelago consists of 4 main islands (Mallorca, Menorca, Ibiza and Formentera) and several uninhabited islets located at the Western Mediterranean Basin. The archipelago constitutes an important nesting place for birds including migratory and sedentary species (Arcos, 2011). Birds included in this study were hospitalized at the Consortium for the Recovery of Wildlife of the Balearic Islands (herein COFIB hospital), between 2010 and 2022. Most birds were admitted due to injuries caused by electrocution, cranial trauma or collisions with cars or fences. Necropsies were performed at the COFIB hospital on dead or euthanized individuals. Among the birds with air sac nematodes, some specimens were collected manually and stored in 70% alcohol for further investigations.

### Morphological identification of parasites

Morphological identification was conducted using both, light and scanning electron microscopy (SEM). Specimens were slide-mounted in Amann lactophenol and subsequently examined and identified under a light microscope (Euromex iScope, The Netherlands) at 100× and 400× magnifications. A morphometric analysis was conducted in well-preserved specimens (26 females and 11 males). The analysis included measurements of the total body length and maximum width, the length of the oesophagus, the length of spicules (for males), as well as measurements of eggs, ovejector and distance from the vulva to the anterior end (for females). Parasites were identified using generic and specific dichotomous keys and species descriptions (Skrjabin and Schikhobalova, 1936; Anderson and Chabaud, 1958; Chabaud *et al*., 1959; Anderson and Bain, 1976).

Some specimens (5 males and 3 females) were preserved for SEM examination to better observe characters which are often difficult to visualize with light microscopy examination. To achieve this, the nematodes initially fixed in 70% ethanol underwent dehydration in an ethanol series and critical point drying with carbon dioxide in an Emitech K850X (Quorum Technologies Ltd., Laughton, East Sussex, UK). Subsequently, the specimens were mounted on stubs with conductive adhesive tape and colloidal silver, coated with carbon in an Emitech K950X (Quorum Technologies Ltd.) evaporator, and examined using a field emission SEM JSM-7001F (Jeol Ltd., Tokyo, Japan) at 10 kV in the ‘*Centres Científics i Tecnològics’ of the University of Barcelona'* (CCiTUB).

### Molecular characterization

Eleven nematodes (including males and females) were randomly selected and stored in 96% ethanol, at −20°C for further molecular analysis. These belonged to the 3 affected strigiform bird species hospitalized in different years (2016, 2018 and 2021). Whole specimens were processed using the NZY Tissue gDNA Isolation Kit (Nzytech, Lisboa, Portugal). The specifications of the manufacturer were followed except for the pre-lysis step, where samples were incubated 24 h at 56°C instead of 1–3 h as specified, and the final elution step, where only 40 μL elution buffer was used instead of 100 μL to increase DNA yield. Molecular characterization was conducted in at least 1 nematode per bird species and year, when possible.

DNA obtained was estimated through a NanoDrop 2000 spectrophotometer (Thermo Scientific, Wilmington, USA). We amplified and sequenced the cytochrome c oxidase I (COI) barcode region and the small ribosomal subunit (18S). The polymerase chain reaction (PCR) was performed using a Veriti Thermal Cycler (Applied Biosystems, Foster City, USA). For the COI amplification, PCR was performed using a pair of primers LCO1490: 5′-GGTCAACAAATCATAAAGATATTGG-3′, and HCO2198: 5′-TAAACTTCAGGGTGACCAAAAAATCA-3′ (Folmer *et al*., 1994), which theoretically results in a 710 bp amplicon. If amplification was not possible, we used the following primers: COI F: 5′-TTTTTTGGGCATCCTGAGGTTTAT-3′ and COI R: 5′-TAAAGAAAGAACATAATGAAAATG-3′ (Monte *et al*., 2012), in which case, the resulting amplicon is a 394 bp fragment. For the 18S region, the primers NEMFG1: 5′-TCTCCGATTGATTCTGTCGGCGATTATATG-3′ and CRYPTOR: 5′-GCTTGATCCTTCTGCAGGTTCACCTAC-3′ (Bimi *et al*., 2005) were used, which theoretically results in an amplicon around 1700 bp.

The reactions were carried out in a final volume of 50 μL. The PCR mix contained 2 μL 10 μm of each primer, 2 μL of the sample's DNA, 25 μL of the Supreme NZYtaq II 2× Green Master Mix (Nzytech, Lisboa, Portugal), 17 μL of MilliQ water and 2 μL of MgCl_2_ 50 mm. For the COI region amplification, PCR was performed under the following conditions: initial denaturation step at 95°C for 3 min followed by 35 cycles at 95°C for 30 s, 50°C for 30 s, 72°C for 1 min and a final extension step at 72°C for 10 min. For the 18S region, the PCR was performed with an initial denaturation step at 95°C for 15 min followed by 35 cycles at 94°C for 30 s, 60°C for 30 s, 72°C for 90 s and a final extension step at 72°C for 10 min.

PCR products were then visualized on a 2% agarose gel stained with Pronasafe nucleic acid solution (Conda Laboratories, Madrid, Spain). Before sequencing, PCR products were purified using the NZY Gelpure kit (Nzytech, Lisboa, Portugal) according to the manufacturer's instructions except for the final step, where the samples were eluted in 30 μL MilliQ water instead of 50 μL elution buffer. Purified PCR products were sent to Sistemas Genómicos S.L. (Spain) and Macrogen (Spain) for bi-directional Sanger sequencing.

Final sequences were uploaded to GenBank and subjected individually for BLAST searches in the NCBI database (https://blast.ncbi.nlm.nih.gov). For phylogenetic reconstruction we retrieved the first 100 hits and manually added sequences from other members of the order Spirurida. All sequences were aligned using MUSCLE (Edgar, 2004) and manually checked for inconsistences. Phylogenetic trees based on maximum-likelihood were constructed for both COI and 18S sequences using MEGA11 software (Tamura *et al*., 2021). The best substitution models (Kimura 2-parameter + G + I and Tamura–Nei + G for 18S and COI, respectively) were determined using MEGA 11 software. A bootstrap test with 1000 replicates was used to determine robustness. Sequences of the trichurid *Trichuris trichiura* (Linnaeus, 1771) were included in the analysis as an outgroup, in both COI and 18S trees.

### Prevalence trends

Statistical analyses were conducted with R software version 4.3.2 (R Core Team, 2023). The prevalence for each host species and year was calculated as the percentage of positive necropsies over the total number of necropsies conducted. Prevalence was calculated for the whole period (2010–2022) as well as for each year. Prevalence trends were plotted with the ‘ggplot2’ R package (Wickham, 2016). Prevalences 95% confidence intervals (CIs) were calculated by the Clopper–Pearson method using the ‘binom’ R package (Dorai-Raj, 2022). As we considered each bird species independently, the years previous to the first detection (before 2014) were removed from the analyses. To evaluate the trend of infection, a generalized linear model (GLM) per host was run with the raw output of each necropsy (1 = infected; 0 = not infected). A binomial distribution with link logit was used as a family. The variable *Year* was used as a predictor of infection. The models were evaluated with the ‘DHARMa’ R package (Hartig, 2022). A variable was considered statistically significant when the *P* value was ⩽0.05. The data and the code used in the analysis can be found in the Supplementary materials.

## Results

### Host data

In total, 641 Strigiformes belonging to 3 species (173 Eurasian scops owls, *Otus scops* (Linnaeus, 1758); 212 long-eared owls, *Asio otus* (Linnaeus, 1758) and 256 barn owls, *Tyto alba* (Scopoli, 1769)) were necropsied at the COFIB hospital between 2010 and 2022. The 3 bird species are listed as Least Concern according to the IUCN Red List of Threatened Species with a decreasing global population trend in all but *T. alba*, which keeps a stable global population trend (BirdLife International, 2019, 2021*a*, 2021*b*). The owls entered the COFIB hospital due to different causes; the most typical being collisions with vehicles, collisions with fences, malnutrition or unknown trauma, among others. The birds necropsied were the ones for which rehabilitation was not possible. No reports of air sac nematodes were reported in any bird species prior to 2014.

### Morphological identification of the parasites

**Figure 1.**
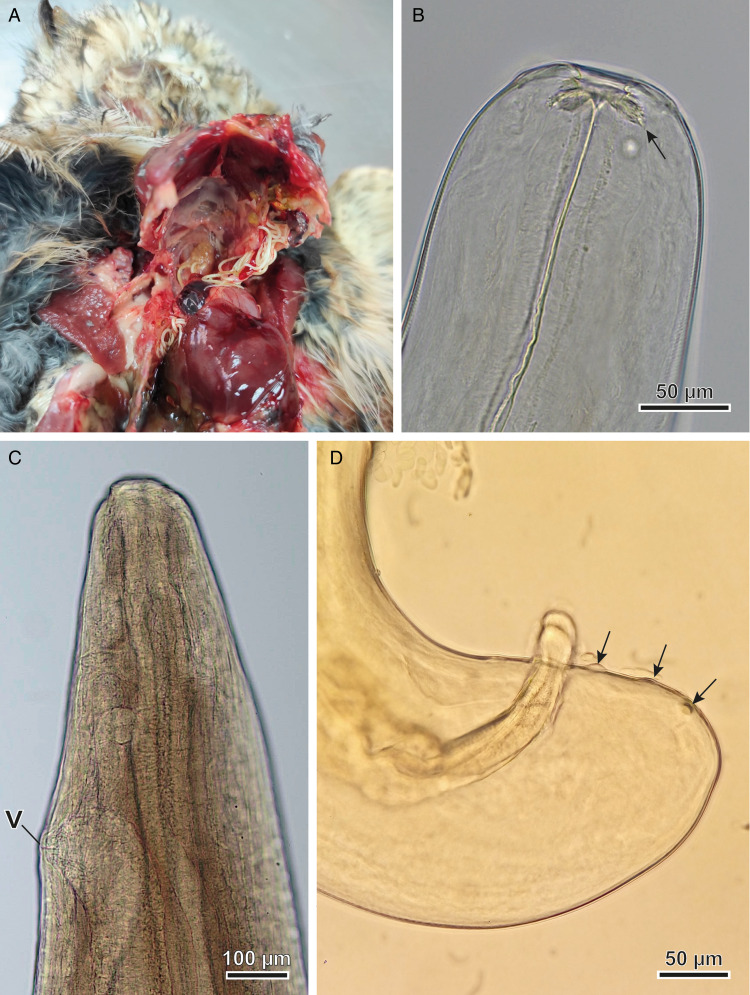
Photographs of *Lissonema sicki* found infecting Strigiformes in this study. (A) Nematodes found free mainly in the thoracic and clavicular air sacs during necropsies. (B) Anterior end of *L. sicki*, showing the buccal capsule (arrow). (C) Female with the vulva (v) near the cephalic end. (D) Male caudal region. Note the 2 almost equal spicules. Arrows indicate the 3 pairs of postcloacal papillae characteristic of this species.

**Figure 2.**
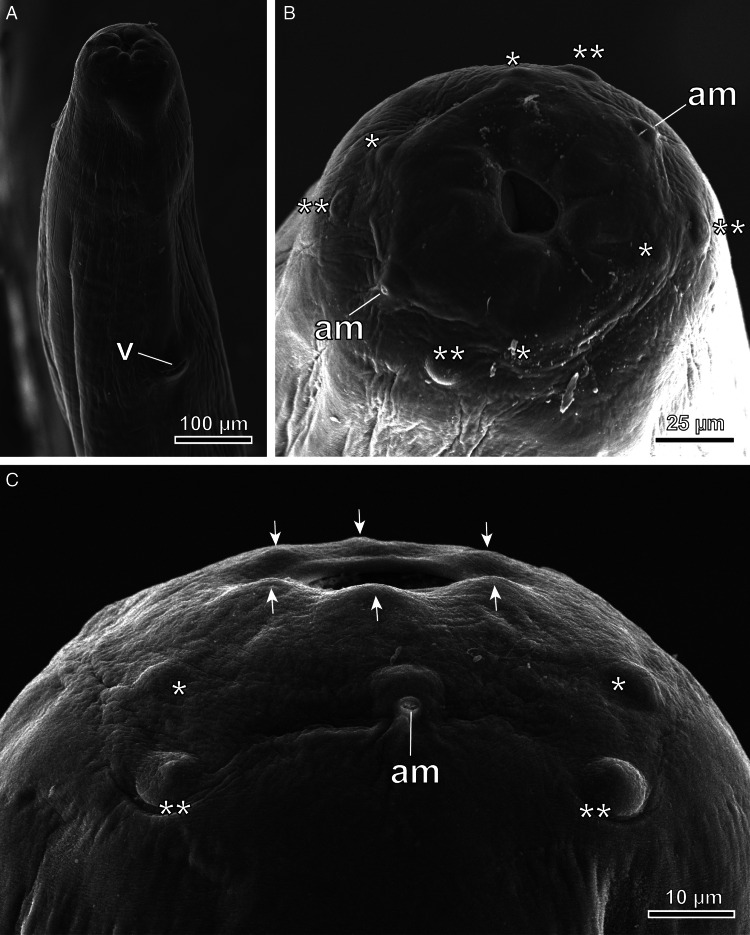
SEM of the anterior end of *L. sicki* (A and B, female; C, male). v, vulva; am, amphids; *, **, anterior and posterior papillae of the 4 pairs of cephalic papillae; arrows, 2 trilobed lateral elevations.

**Figure 3.**
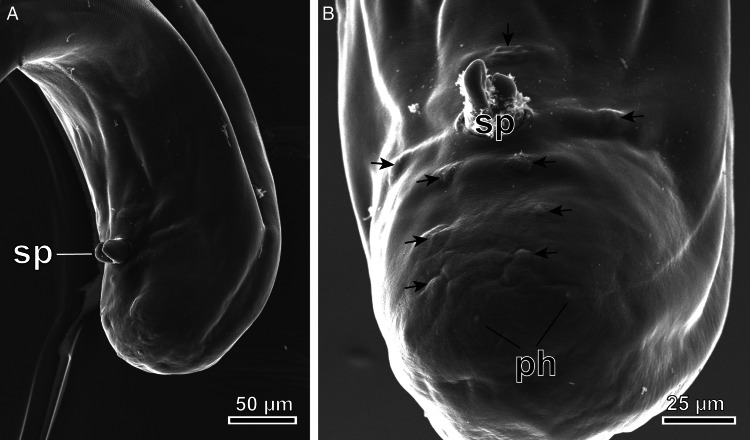
SEM of the posterior end of a male of *L. sicki*. (A) General view of the posterior end; (B) details showing the distribution of cloacal papillae in ventral view. sp, spicules; ph, phasmids; arrows, cloacal papillae.

The nematodes (Fig. 1A) found in the air sacs of *A. otus*, *O. scops* and *T. alba* were morphologically identified as *Lissonema sicki* (Strachan, 1957) (Aproctidae). The nematodes were found free mainly in the thoracic and clavicular air sacs. The taxonomy of *L. sicki* is complex as many synonyms exist in the literature. This taxon was first described as *Thelazia sicki* by Strachan (1957) and later included in the genus *Squamofilaria* Schmerling, 1925, following a redescription of the species by Anderson and Chabaud (1958). In the present study, we adhered to the generic diagnosis provided by Bain and Mawson (1981), in which *Squamofilaria sicki* was classified within the genus *Lissonema* Linstow, 1903, a group known to have Strigiformes as their definitive hosts. The present specimens possessed a funnel-shaped buccal capsule and an oesophagus with no differentiation between the muscular and glandular regions (Fig. 1B). The cephalic end had 2 trilobed elevations, 4 pairs of cephalic papillae (the posterior papilla of each pair was bigger) and a pair of amphids (Fig. 2B and C). The morphometric analysis showed that female nematodes were larger, being 29.12 ± 3.8 mm long and 559 ± 96 μm width (Table 1), with a well-defined vulva, located near the anterior end (Figs 1C and 2A). Thick-shelled eggs were observed in all female specimens. Male specimens were 12.54 ± 1.5 mm long and 370 ± 84 μm wide, with a short, curved and rounded tail. The spicules were equal in length and shape (Table 1). The proximal extremity of the spicules was massive and the distal extremity bluntly rounded, flattened and membranous (Figs 1D and 3A). Males exhibited 3 pairs of postcloacal papillae (not always visible with a light microscope), 1 pair of precloacal papillae situated near to the anterior margin of the cloaca and an unpaired precloacal papilla (Figs 1D and 3B). The 2 phasmids were located posterior to the last pair of postcloacal papillae (Fig. 3B). All morphoanatomical features observed in our specimens matched those redescribed for *L. sicki* by Anderson and Chabaud (1958).

**Table 1.** Comparative morphometry of the air sac nematode *Lissonema sicki* found in the Strigiformes of this study *vs* the original redescription and the morphometry of *Lissonema noctuae* (a closely related species).

	*L. sicki*		*L. noctuae*		
	This study	Anderson and Chabaud (1958)	Sonin (1966)	Yoshino *et al*. (2014)	Zhang *et al*. (2012)
Male (*n*)	11	1	?	?	2
Body length	10 000–15 000	13 000	16 000	11 540	1262–1310
Body width	237–492	390	480	450	440–490
Oesophagus length	799–1099	870	1050	980	1260–1360
Nerve ring	–	160	190	130	130–150
Excretory pore	–	200	–	–	320–340
Right spicule length	170–187	210	160	160	160
Left spicule length	170–187	200	160	160	170
Precloacal papillae (pair)	1 + 1 single	1 + 1 single	–	–	1
Postcloacal papillae (pair)	3 + 1 phasmid	3 + 1 phasmid	–	–	1
Female (*n*)	26	1	?	?	5
Body length	18 000–35 000	19 000	43 000	27 470	24 760–27 870
Body width	377–818	520	650	730	730–780
Oesophagus length	1012–1416	880	1340	1190	1260–1460
Nerve ring	–	150	170	150	170–220
Excretory pore	–	–	–	–	280–290
Distance vulva–anterior end	376–741	430	750	600	520–640
Ovejector length	651–1688	–	–	–	–
Egg length × width	58–70 × 28–40	66 × 33	70–78 × 25–40	68–78 × 32–36	70–80 × 30–40
Host	*Otus scops*, *Asio otus*, *Tyto alba*	*Otus* sp.	*Athene noctua*, *Otus sunia*	*O. sunia*	*O. scops*
Country	Spain (Mallorca)	Brazil	Far East, Morocco	Japan	China

All measurements are given in μm.

### Molecular characterization

We obtained 11 COI and 4 18S sequences from individual specimens. In the case of COI, none of the specimens could be amplified with primers by Folmer *et al*. (1994); however, they were successfully amplified and sequenced using the primers developed by Monte *et al*. (2012). All sequences were submitted to GenBank with the following accession numbers: OQ941465–OQ941475 (COI sequences) and PP838209–PP838212 (18S sequences). The resulting alignments were 355–394 and 1539 bp in length, for COI and 18S respectively. The BLAST resulted with our sequences being >12 and >7% distant with the closest ones for COI and 18S, respectively, therefore, species identity could not be determined using this approach.

**Figure 4.**
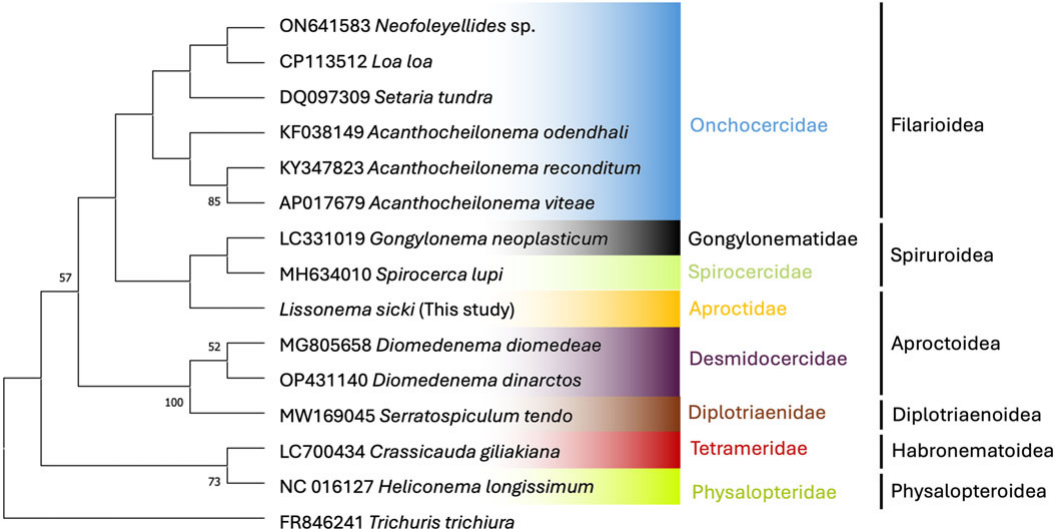
Maximum-likelihood phylogenetic tree inferred from COI sequences showing the position of *L. sicki* in relation to other species of the order Spirurida. Bootstrap values (1000 replicates) lower than 50 are not shown.

**Figure 5.**
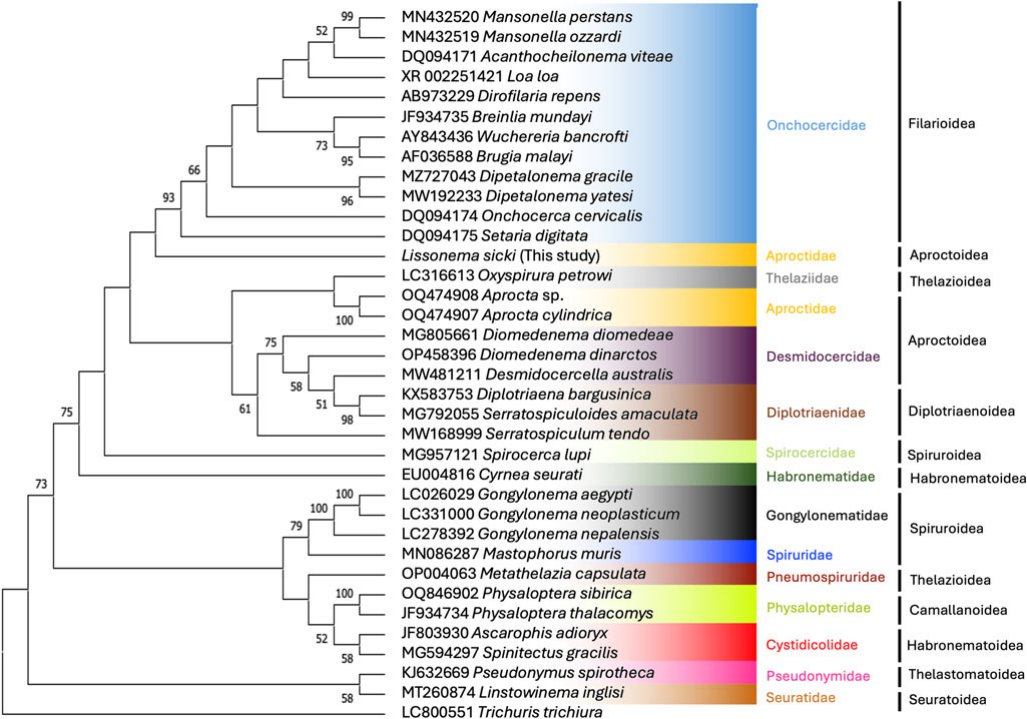
Maximum-likelihood phylogenetic tree inferred from the 18S sequences showing the position of *L. sicki* in relation to other species of the order Spirurida. Bootstrap values (1000 replicates) lower than 50 are not shown.

Both phylogenetic trees place *L. sicki* within the Spirurida (Figs 4 and 5). However, *L. sicki* does not appear in the Aproctidae clade (represented by members of the *Aprocta* genus). Instead, it appears in the same clade as members of the Onchocercidae family (bootstrap support <50%) in the 18S tree (Fig. 5). The phylogenetic tree using the COI gene region (Fig. 4) is less informative because sequences of other members of the Aproctidae could not be retrieved from GenBank. Furthermore, the number of sequences from representatives of the Spirurida was limited for this gene. Bootstrap values were low in both cases.

### Trends in prevalence and clinical information

**Figure 6.**
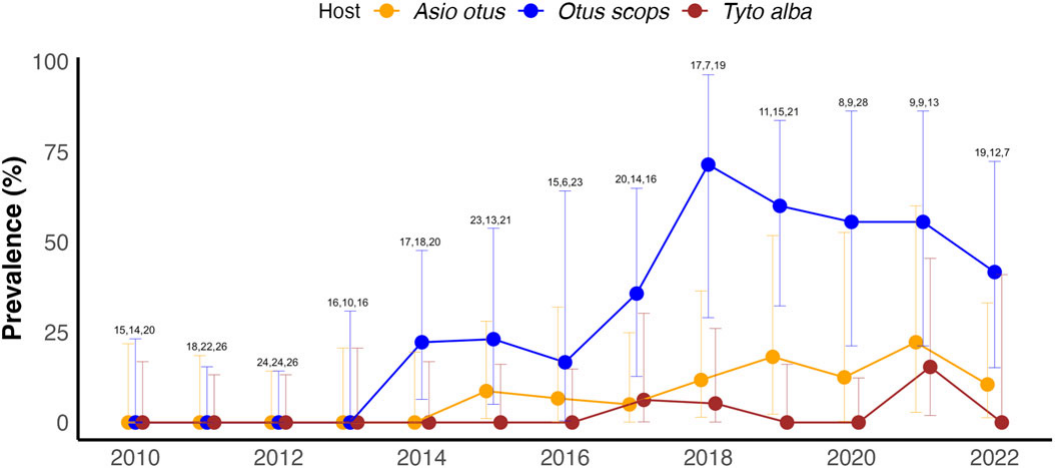
Prevalence of *L. sicki* in necropsied Strigiformes per year during this study (2010–2022). Graphic created with ‘ggplot2’ R package. Error bars represent the 95% CIs according to the Clopper–Pearson method. Numbers on the upper error bars show the total number of birds necropsied per year and species (*Asio otus*, *Otus scops* and *Tyto alba*, respectively).

The first detection of the air sac nematode *L. sicki* occurred in 2014 in *O. scops*, while the detections in *A. otus* and *T. alba* occurred in 2015 and 2017, respectively. Since then, the prevalence of *L. sicki* has increased in all 3 species, reaching its maximum peak in 2018 in *O. scops* and showing a slight decrease in 2022 in all hosts. Throughout the period of the first detection of the nematode to 2022, the overall prevalence of air sac nematodes on necropsied owls was 40.8% (CI 31.2–50.9%) in *O. scops*, 9.3% (CI 5.1–15.5%) in *A. otus* and 2.4% (CI 0.6–6%) in *T. alba* (Fig. 6). The GLM models indicate a significantly increasing trend in prevalence only in *O. scops* (GLM, *P* value = 0.014, slope = 1.21), while in the rest of the bird species this increment was not statistically significant (*A. otus* GLM, *P* value = 0.120, slope = 1.18; *T. alba* GLM, *P* value = 0.193, slope = 1.36). Slope is presented as the *e*^estimate^.

All infected birds were adults, except for 2 juvenile *O. scops* specimens. Parasite intensities ranged from 2 to >80 nematodes per infected bird. As the examined birds were hospitalized and later died due to severe injuries (such as collisions or fractures), the clinical severity related to the presence of air sac nematodes could not be assessed.

## Discussion

In recent years, air sac helminths have been reported as emerging pathogens of birds of prey in various regions worldwide (Gomez-Puerta *et al*., 2014; Ibarra *et al*., 2019). Although most infections are typically mild, severe and fatal cases caused by these parasites have been documented (Galosi *et al*., 2019; Díaz *et al*., 2022). This study introduces *L. sicki*, an air sac nematode that since its initial detection in the island of Mallorca in 2014 has shown an increase in prevalence among both mostly sedentary (*A. otus* and *T. alba*) and migratory (*O. scops*) owls on the island. Although sporadically documented only in South America, the first detection of *L. sicki* in Europe has prompted a comprehensive characterization of its morphology, molecular identity and ultrastructure to facilitate future detections and enhance our understanding of the impact *L. sicki* has on susceptible hosts.

The members of the Aproctidae are known to be parasites inhabiting the cervical air sacs, nasal cavities, orbits and incidentally the subcutaneous tissue of birds (Anderson and Bain, 1976; Anderson, 2000). The taxonomy of the members of this family remains poorly explored (Vanstreels *et al*., 2018). In this context, the identification of our specimens was challenging and laborious. Light microscopy showed that our specimens' morphology matched those of the South American parasite *L. sicki* and *Lissonema noctuae* (Spaul, 1927), a species found in North Africa (Spaul, 1927), Japan (Yoshino *et al*., 2014) and China (Zhang *et al*., 2012). The differentiation between *L. sicki* and *L. noctuae* relied mainly on the number and arrangement of postcloacal papillae [3 pairs in *L. sicki* (Anderson and Chabaud, 1958) and from 0 to 1 pair in *L. noctuae* (Spaul, 1927; Zhang *et al*., 2012; Yoshino *et al*., 2014)], which are difficult to differentiate with light microscopy (Table 1). However, based on SEM analysis, we were able to confirm the presence of 3 postcloacal pairs of papillae in the present specimens (Fig. 3). Other members of the genus were not considered given their different morphology and host range.

This study provides the first molecular characterization (18S and COI) of *L. sicki*, potentially contributing to the early detection of this species in other regions. Additionally, it increases the dataset for the Aproctidae, given that so far, only 1 previous study has molecularly characterized the members of this family (Niedringhaus *et al*., 2023).

Despite the low bootstrap values obtained, which prevent drawing firm conclusions, the phylogenetic reconstruction of members of the Spirurida using the 18S gene suggests that *L. sicki* may be more closely related to Onchocercidae than previously stated (Fig. 5). However, as previously reported for this order, the limited number of available sequences may provide misleading results and should be interpreted cautiously (Nadler *et al*., 2007). According to Bain *et al*. (2014), morphological characters in adults of the order Spirurida have little phylogenetic value because they are formed late during development and are thus subject to homoplasy. We strongly recommend carrying out further molecular-based studies to not only clarify the taxonomic position of *L. sicki* but also that of other taxa within this order.

This study represents the first report of *L. sicki* in Europe, outside of its little known native Neotropical region. Before 2014, no air sac nematode species had been observed in Mallorcan strigiform birds. To our knowledge, *L. sicki* had only been previously reported in Brazil, where it was found infecting the ocular region of an unidentified *Asio* sp. (Strachan, 1957) and later in Bolivia (Garvin *et al*., 1997), infecting the nasal cavity of an *Otus choliba* (Vieillot, 1817). Similar to other members of the superfamily Aproctoidea, *L. sicki* is likely transmitted through the ingestion of insects of the orders Orthoptera or Coleoptera (Anderson and Bain, 1976; Anderson, 2000), which are known to play a crucial role in the diets of *O. scops* (Marchesi and Sergio, 2005), *T. alba* (De Pablo, 2000) and *A. otus* (Trujillo *et al*., 1989).

**Figure 7.**
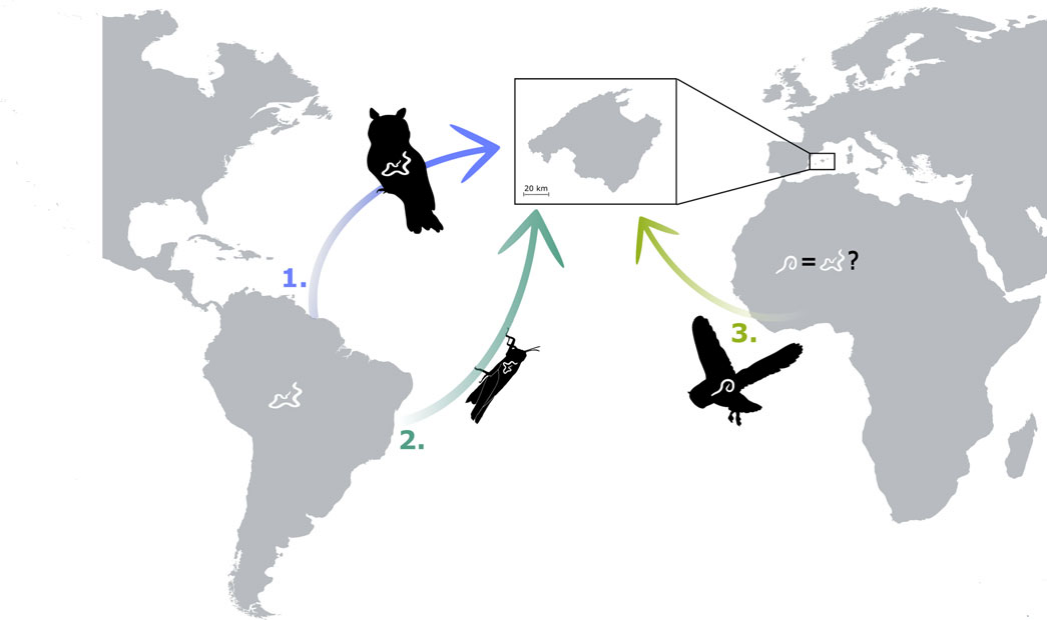
Schematic figure representing the possible entry routes of *L. sicki* to Mallorca (Balearic Islands, Spain). (1) Introduction through illegally traded infected birds, (2) co-introduction with invasive intermediate hosts and (3) introduction through North African birds *via* migratory routes if *L. sicki* is a synonym of *Lissonema noctuae*.

We hypothesize that the invasion of *L. sicki* may have occurred through at least 3 possible routes: (1) introduction through illegally traded infected birds, (2) co-introduction with invasive intermediate hosts and (3) introduction through North African birds *via* migratory routes (Fig. 7). The first hypothesis is grounded in the occurrence of the illegal trade of *O. choliba*, its host in South America. This bird species ranks among the most trafficked bird species in South American countries (WCS Colombia, 2021). Previous studies have demonstrated that trafficked birds harbour parasites, and due to their compromised immune status, they often harbour high parasitic burdens (Arbetman *et al*., 2013). Despite this, to the best of our knowledge, there are no reports of the introduction of *O. choliba* specimens in Europe. However, it is worth noticing that some eggs from this species were confiscated in 2008 in Portugal (Ortiz-von Halle, 2018). The second hypothesis is rooted in the establishment of several insect species in the Balearic Islands (Torres *et al*., 2021). The phenomenon of insects carrying pathogens from their native range is well-documented (Vilcinskas, 2019), with some native host populations from invaded territories showing higher infection rates than the invasive hosts (Arbetman *et al*., 2013). Finally, the difficulties observed in differentiating *Lissonema* species using light microscopy, coupled with the scarcity of new studies in these species may imply that *L. noctuae* from North Africa could be conspecific with *L. sicki*. This would imply *L. sicki* to be a synonym of the first described *L. noctuae*. However, further molecular studies comparing samples of both species and other members of this family should be performed to clarify the taxonomy of this group of nematodes. Notably, this region is part of the Mediterranean migration route of *O. scops* (Barriocanal *et al*., 2021). Migratory birds are known to harbour a wide variety of parasites, and the close phylogenetic relationships between hosts facilitate cross-species transmission (Ishtiaq and Renner, 2020).

The increase in *L. sicki* prevalence observed in Mallorcan owls, reaching peaks of 71.43% in *O. scops*, 22.22% in *A. otus* and 15.38% in *T. alba*, shows that the Mediterranean basin has optimal conditions for the completion of the life cycle of this parasite. It is worth noting that data on prevalence presented in this study should be interpreted carefully, as it pertains solely to necropsied birds from the COFIB hospital and may not represent the real prevalence of the parasite in nature. Santoro *et al*. (2010) reported a high (80.6%) prevalence of the air sac nematode *S. tendo* in *F. peregrinus* in Italy with a higher severity of the clinical signs been directly related to parasite load. The high infection burden (>80 nematodes/host) reported in this study may indicate that *L. sicki* has the potential to severely impair birds' fitness. The only 2 reports of *L. noctuae* indicate that this parasite causes low parasitaemia, with only 1–3 nematodes per host (Spaul, 1927; Yoshino *et al*., 2014). Given that air sac nematodes have been associated with lethal infections in birds (Lavoie *et al*., 1999; Samour and Naldo, 2001), further studies should be conducted to assess the detrimental effect that *L. sicki* could have on owls.

The Eurasian scops owl, *O. scops*, is the only European species undertaking a lengthy migratory route, overwintering in the South of the Sahara Desert (Barriocanal *et al*., 2021). The significantly higher prevalence observed in *O. scops*, in comparison to other owl species from Mallorca, may indicate a higher specificity of *L. sicki* for *O. scops*. Given that *O. scops* is present in various parts of the continent, the spread of the parasite to other regions is likely to occur. In this context, Mediterranean islands have served as gateways for several emerging parasitic helminths in Europe, subsequently reported in continental territories (Salas-Coronas *et al*., 2021; Paredes-Esquivel *et al*., 2023). We speculate whether islands can be used for the early detection of emerging pathogens on the continent, given their susceptibility to the establishment of invasive species (Reaser *et al*., 2007). Under the current scenario of global change, vigilance over emerging pathogens that may affect bird populations is imperative, as it has a direct influence on phenomena such as host switching and trophic dynamics.

## Supporting information

Jaume-Ramis et al. supplementary material 1 Jaume-Ramis et al. supplementary material

Jaume-Ramis et al. supplementary material 2 Jaume-Ramis et al. supplementary material

## Data Availability

The authors confirm that the data supporting the findings of this study are available within the article (and/or its supplementary materials).

## Supplementary material

The supplementary material for this article can be found at https://doi.org/10.1017/S0031182024000805.
